# Inflammation in the Transition Period of Dairy Cows: New Paradigms

**DOI:** 10.3390/vetsci12121182

**Published:** 2025-12-10

**Authors:** Alexandro Fritzen, Aleksandro Schafer da Silva

**Affiliations:** 1 Multicenter Postgraduate Program in Biochemistry and Molecular Biology, Santa Catarina State University (UDESC), Lages 88520-000, Brazil; 2 Department of Animal Science, Santa Catarina State University (UDESC), Chapecó 89815-630, Brazil

**Keywords:** transition, dairy cows, immunometabolism, immune dysregulation, inflammatory resolution

## Abstract

By analyzing new findings from the literature on immunometabolism during the transition period, as well as classical concepts, we aim to propose a new approach to the inflammation paradigm in cows transitioning from gestation to lactation. The purpose of this review, based on data from scientific articles and reviews, is to characterize the immunometabolism of the transition period, highlighting the main characteristics of the inflammatory response in dairy cows and listing the mechanisms that govern inflammatory tone and coordinate the resolution of inflammation, with a view of new paradigms and analysis of non-classical pathways. We also seek to analyze inflammation and the role of mitochondria and the endoplasmic reticulum in the genesis of diseases, proposing approaches for modulating inflammatory tone. This review analyzes the classical theoretical approach using mechanistic views anchored in knowledge produced in other species and based on new evidence from the bovine field, determining new paradigms. This study highlights the constant inflammatory condition during the transition period and its effect on metabolism and demonstrates the role of dysregulation of inflammation resolution as a central factor in the genesis of diseases. Purinergic and cholinergic anti-inflammatory pathways have been little explored in cattle, but it is believed that the role of these pathways in maintaining homeostasis represents a great opportunity for understanding and modulating the phenomenon of inflammation in dairy cows. Dysregulation of the stress axis shows elucidative potential regarding the dysregulation of inflammatory resolution, with mitochondrial health and endoplasmic reticulum stress being dividing factors between homeorhetic and pathological states. Modulating inflammation and establishing inflammatory resolution presents challenges with classic anti-inflammatory drugs due to the paradoxical role of prostaglandin E2 in inflammation and resolution. Phytoactives offer new possibilities for modulating inflammation without disrupting inflammatory resolution and ensuring antioxidant effects, associated with strategies for formulating less inflammatory diets.

## Simple Summary

The inflammatory state is constant in dairy cows and predisposes them to the development of diseases, with some paradigms being addressed in this review: (a) the involvement of axes such as GH/IGF-1 and ACTH/cortisol in the regulation of the inflammatory process; (b) modulation of the tissue macrophage profile, which demonstrates great potential in controlling inflammation, lipolysis, and improving energy metabolism; (c) the integrity of mitochondria and the endoplasmic reticulum, which have roles in maintaining animal health; and (d) the role of nutrition and omega 3 and 6 levels together with diets that are more friendly to gastrointestinal health, with additives such as choline, as factors in the modulation of immune health via the cholinergic system.

## 1. Introduction

The inflammatory process in dairy cows has gained notoriety due to its association with the development of transition diseases [1], dividing opinions and redefining what we consider normal for high-performance dairy cows [2,3]. This review investigates the origin and regulation of inflammation during the transition period in dairy cows, analyzing its neuroendocrine modulation and its interaction with metabolism, proposing new paradigms for the classical interpretation of this topic, and addressing the hypothesis of dysregulation of inflammatory resolution in dairy cows. We also analyzed non-classical pathways of inflammatory regulation, such as the purinergic and cholinergic systems and their role in regulating inflammatory resolution in dairy cows. By addressing biochemical and molecular concepts, we aim to present a review that contributes a focused view of the genesis of immunometabolism dysregulation during the transition, reviewing the regulation of inflammatory resolution, with the objective of exposing pathways involved in inflammatory dysregulation, accompanied by mitigating approaches to the problem.

## 2. Immunity Characterization of the Transition Period of Dairy Cows

**Figure 1:**
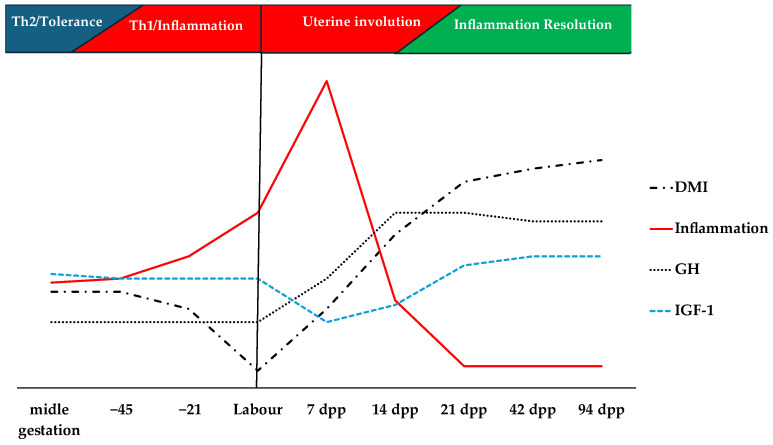
Diagram of the behavior of dry matter intake (DMI), inflammation, growth hormone (GH), and insulin-like growth factor-1 (IGF-1) during pregnancy, labor, and postpartum. The final phase of pregnancy is marked by a shift from Th2 to Th1 immune response, leading to increased immunoreactivity at the end of gestation, accompanied by a reduction in dry matter intake (DMI) and increased inflammation. In the immediate postpartum period, dry matter intake is low and gradually increases, while GH levels progressively rise. A reduction in IGF-1 levels occurs postpartum and is associated with the uncoupling of the somatic–tropic axis, with a progressive increase to normal levels. Postpartum, there is a marked increase in inflammation in the first week, with a progressive reduction to normal levels in the subsequent period.

The final phase of gestation is marked by reduced immunotolerance to fetal tissues and a shift from a helper Th2 response to a Th1 response in the postpartum period [4], as shown in Figure 1. Favero et al. [5] demonstrated that the initial and middle third of gestation in cattle is accompanied by reduced levels of tumor necrosis factor alpha (TNF-α), Interleukin-6 (IL-6), adenosine deaminase (ADA), and reactive oxygen species (ROS), when compared to non-pregnant cows. However, with the increased immunoreactivity of fetal tissues, an increase in pro-inflammatory cytokines is observed, accompanied by an acute phase response and increased oxidative stress [5,6], which comprises a tolerance period. The last twenty-one days of gestation are accompanied by an increase in positive acute phase proteins and reactive oxygen species, with a reduction in dry matter consumption (Figure 1) [6,7], and the beginning of the mobilization of body reserves, characterizing the emergence of the negative energy balance (NEB) [8,9], demonstrating a natural increase in inflammatory tone in the final period of pregnancy. The increase in inflammatory cytokines is closely related to the occurrence of postpartum diseases [10] and is possibly responsible for the suppression of the observed dry matter intake (DMI) [11]. In this context, we believe that the exposure of cows to the inflammatory state is constant, and the relationship between cytokines and diseases demonstrates that the activation of the immune system is involved in the genesis of transition diseases; and that this correlation does not rule out the possibility of confusion, since all cows are inflamed, but only a portion develops the disease.

The transition period is marked by a reduction in insulin levels and tissue responsiveness of this hormone, with an increase in growth hormone (GH) levels and a reduction in IGF-1, characterizing uncoupling of the somatotropic axis and peripheral insulin resistance (Figure 1) [12,13,14,15]. This condition prioritizes the flow of nutrients to the mammary gland, supporting lactation, but leads to mobilization of body reserves with a consequent increase in non-esterified fatty acid (NEFA) levels and ketogenesis [16], but the intensity of this phenomenon is variable from cow to cow. With the onset of labor, an increase in blood glucose levels is observed, but after the start of lactation, glucose levels decrease considerably, with an increase in triglyceride accumulation in hepatocytes [7,17]. These metabolic states are common to homeorhetic metabolism, being disturbed, in our view, by the maintenance of concomitant chronic inflammation, which compromises the ability to control the uncoupling of the somatotropic axis and lipolysis.

The labor and onset of lactation is marked by a reduction in serum calcium levels, with varying levels of hypocalcemia with an impact on gastrointestinal motility, and consequent interference in feed intake, increasing the mobilization of body reserves and loss of body condition [18]. The relationship between calcium levels and the activation of the immune system has been proposed [3], and it has been observed that the reduction of calcium levels increases the activity of ADA, an enzyme responsible for the deamination of adenosine, a molecule with anti-inflammatory effects [19] involved in resolution of inflammatory responses in human [20]. The relationship between failure of inflammatory resolution and hypocalcemia is a topic to be explored in postpartum cows.

In cattle, the reduction in progesterone levels and the beginning of parturition is associated with the release of fetal cortisol [21], because this period is accompanied by a change in the responsiveness of the cow’s hypothalamic–pituitary–adrenal axis, with an increase in basal levels and loss of the pulsatile pattern of release of cortisol, which is also observed in models of immune activation by lipopolysaccharide (LPS) and insulin resistance [22,23]. The increase in NEFA, β-hydroxybutyrate (BHB) levels, and inflammation in late gestation and lactation reduces adrenal response to adrenocorticotropic hormone (ACTH) stimulation with lower cortisol release [24]. Glucocorticoids act to regulate the expression of GH receptors (GHr) in the liver, reducing GHr and decreasing circulating IGF-1 levels [25], an effect that is reversed by insulin [12]. The reduction in responsiveness and sensitivity to cortisol and insulin associated with the reduction in IGF-1 are observed as conditions of homeorhetic regulation [14], but it needs to be clear that the intensity of the reduction in sensitivity and responsiveness together with the time for their normalization in the postpartum period depends on individual factors, with the limit between homeorhetic, and pathological being unclear.

During the first weeks of lactation, increased levels of GH and cortisol and reduced insulin sensitivity stimulate the activation of HSL (hormone-sensitive lipase) in adipose tissue, releasing NEFAs into the circulation with concomitant changes in lipidemia [26]. NEFAs can be taken up by the mammary gland and incorporated into the synthesis of milk fat or be taken up by the liver where they are oxidized to generate energy [27]. However, in the postpartum period, low glucose availability leads to β-oxidation, and an increase in Acyl-CoA, which predisposes to ketogenesis [28,29] and production of ROS [30], a condition that is intensified by the reduction in glucose availability promoted by glycolysis associated with inflammation. The remodeling caused by lipolysis in adipose tissue is associated with the infiltration of macrophages into adipose tissue, being greater in cows with intense fat mobilization [31], which in animals, with ketosis and abomasum displacement, is associated with the polarization of macrophages into an M1 phenotype with inflammatory activity and production of IL-1β and TNF-α, increasing local and systemic inflammation [9,32] with a consequent reduction in DMI in a vicious pathological cycle. The effect of macrophage phenotype on the balance between compensated and decompensated lipolysis is still a matter of speculation, but in our opinion, experimental findings in disease models demonstrate a large presence of M1 macrophages in cows with abomasal displacement and ketosis, corroborating the persistence of a sustained inflammatory state, with delay or disruption of inflammatory resolution.

The increased energy demand in the postpartum period leads to strategies such as increasing the energy density of diets by increasing the inclusion of carbohydrates, which may predispose cows to subacute ruminal acidosis and increased production of LPS in the gastrointestinal (GI) tract due to dysbiosis [33,34]. This condition is associated with increased production of ROS in the liver due to increased uptake of LPS from the GI tract via the hepatic portal system [35]. Increased LPS levels induce systemic and adipose tissue inflammation, with reduced insulin sensitivity, that causes lipolysis through the inflammatory pathway with activation of protein kinase A (PKA) and protein kinase C alpha (PKCα), inducing HSL phosphorylation and release of free fatty acids in blood [30].

Lipopolysaccharides may originate from the GI tract, mammary gland, uterus, or other sites of Gram-negative bacteria infection [3,30], but exposure of bovine enterocytes to interferon gamma (INFγ) and TNF-α, in vitro, leads to disruption of intercellular junctions and increased permeability [36], and it is possible that increased cytokines during the transition period disrupt the integrity of the cellular barriers of the gastrointestinal tract, culminating in an increase in circulating LPS, a condition known as leaky gut. Research is needed to elucidate this possible mechanism in vivo, as disruption of intestinal permeability by non-septic inflammatory events may be associated with LPS exposure, leading to confusing cause-and-effect findings.

Lipolysis in postpartum cows is associated with increased synthesis of oxylipins by enzymatic and non-enzymatic pathways that use linoleic acid and arachidonic acid as substrates, producing hydroxy-octadecadienoic acids (HODE) and hydroxy-eicosatetraenoic acid (HETE) with an inflammatory profile [26], as well as increased levels of 13-HODE and 5-, 11-, and 15-HETE observed in the postpartum period [37]. This phase is marked by greater reactivity of mononuclear cells to exposure to LPS with greater production of cytokines compared to animals in mid-lactation [37,38]. The metabolization of 13-HODE to 13-oxooctadecadienoic acid (13-oxoODE), an oxylipin involved in the resolution of inflammation, is performed by nicotinamide adenine dinucleotide phosphate (NADPH)-dependent fatty acid dehydrogenase, which has reduced activity in the postpartum period, leading to an increase in inflammatory oxylipins [37,38]. The increase in energy demand associated with lactation and maintenance of inflammation promotes increased oxidation and production of inflammatory oxylipins, culminating in impaired inflammatory resolution.

We believe that the inflammation observed during the transition is part of the adaptive process, which involves the end of gestation and the onset of milk production. However, the decoupling of the somatotropic axis, accompanied by peripheral insulin resistance and the reduced responsiveness of the hypothalamic–pituitary–adrenal axis, compromises the resolution of inflammation in the postpartum period, keeping the cow inflamed and increasing oxidative stress and energy expenditure. The inability to promptly resolve inflammation, with the consequent increase in oxidative stress, constitutes a paradigm to be addressed in the biology of the transition.

## 3. Inflammatory Response in Dairy Cows

The immune system can recognize DAMPs (damage-associated molecular patterns) and PAMPs (pathogen-associated molecular patterns), generating aseptic and septic inflammation, respectively [39]. These molecules activate pattern recognition receptors (PRRs) in endothelial and epithelial cells, macrophages, and dendritic cells of the innate immune system, leading to the activation of NF-kB (nuclear factor kappa B), and increased expression of inflammatory cytokines, chemokines, and molecules derived from cyclooxygenase and lipoxygenases [4,40].

The activation of toll-like receptors 4 (TLR-4: members of the PRRs) can be carried out by bacterial lipopolysaccharides (LPSs) and by NEFAs such as palmitic acid; however the understanding of the mechanisms of interaction of DAMPs and PAMPs with all PRRs on cells of the bovine immune system is not yet fully elucidated [41,42,43]. Studies with liver tissue of calves demonstrate that high levels of NEFAs induce the production of ROS and activation of NF-kB with its translocation to the nucleus and increased expression of inflammatory cytokines such as TNF-α, IL-1β, and IL-6 [44] characterizing the interaction between lipid metabolism and the innate immune system in cattle.

NLR family pyrin domain containing 3 (NLRP3) inflammasome activation consists of two stages: priming and activation itself [45]. The priming phase is classically activated by the binding of PAMPs or DAMPs to TLR receptors, leading to increased translation and post-translational modifications that prepare NLRP3 for the activation phase. The activation phase requires a second stimulus, such as extracellular ATP, culminating in the activation of caspase-1 and the cleavage of IL-1β into its active form [45,46], with release via pyroptosis, and it is observed that the stimulation of this pathway by BHB in bovine monocytes reduces their phagocytosis capacity but promotes inflammation [47]. For us, this scenario of activation of the innate system leads to increased glucose expenditure, disturbing energy homeostasis and favoring the generation of DAMPs, increasing the support of the pro-inflammatory stimulus, which is counterbalanced by the anti-inflammatory effects of cortisol and IGF-1, together with the regulations promoted by the cholinergic and purinergic systems, but there is no clarity about the role of these systems in maintaining the regulation of the inflammatory process in the transition. The reduction in IGF-1 levels and responsiveness to cortisol favor the disturbance of inflammatory resolution, which points to the difficulty of the cow in modulating and interrupting the natural inflammatory response to the transitory state between pregnancy and lactation.

After activation of PRRs in dendritic cells and macrophages, the neutrophils are recruited to the site of injury along with the processing and presentation of antigens to the cells responsible for the acquired response, such as lymphocytes [4]. With the elimination of the septic focus and clearance of DAMPs, chemokines, and reduction of ROS, the process of neutrophil phagocytosis begins, known as efferocytosis, marking the beginning of the resolution of inflammation and tissue repair [20]; however, these immunological events are unclear in cattle during transition. The polarization of M1, pro-inflammatory macrophages to M2, anti-inflammatory ones is essential for the beginning of the resolution of inflammation, being regulated in humans by the balance of inflammatory and anti-inflammatory oxylipins, vagus nerve activity, IGF-1 levels, and responsiveness of tissues for cortisol [40,43,48,49,50,51]; however, in dairy cows this mechanism is still poorly understood. The poor understanding of the mechanisms of inflammatory resolution associated with the exposure of cows to calving, as well as its corresponding inflammation, leaves questions about the role of inflammatory resolution in the transition, since inflammation is constant.

Macrophage phenotype and its association with pathological states in dairy cows have been gaining attention [43,52,53,54]. Cows with ketosis present macrophages of inflammatory phenotype (M1), leading to the production of ROS and inflammatory cytokines that promote an inflammatory state [54], and increased energy demands for immune system activation [55]. The increased energy demand increases the mobilization of NEFAs and consequently increases the stimulation of innate immune cells via TLR-4, leading to a pro-inflammatory vicious cycle, which is counterbalanced by the action of cortisol, and by the cholinergic and purinergic pathways. The role of these pathways in modulating the macrophage profile in cattle is still unclear considering the current literature. But classical activation of macrophages (M1) influences the way these cells produce adenosine triphosphate (ATP) [49]. In M1 macrophages, the glycolytic pathways are activated with an increase in glycolysis to the detriment of oxidative phosphorylation (OXIPHOS), while the opposite is observed in alternatively activated macrophages (M2), with ATP production centered on oxidative phosphorylation [47,53]. The increase in aerobic glycolysis (warburg effect) increases glucose expenditure with the production of 2 moles of ATP per glucose molecule [3,56]. This increase in glycolysis reduces energy efficiency, since oxidative phosphorylation produces 36 ATP per molecule, totaling 38 ATP per glucose molecule when glycolysis associated with oxidative phosphorylation occurs [56].

The regulation of inflammatory tone significantly influences the way in which dairy cows use energy substrates during the transition period [3,56]. The inflammatory state increases the production of ROS with an increase in the load on redox metabolism, culminating in oxidative stress and disruption of health status [52]. This state demonstrates the constant of inflammation and the importance of controlling inflammatory tone and establishing mechanisms for inflammatory resolution, with the time of exposure to the inflammatory state being a key factor in the success of adaptation to lactation.

### Regulation of Inflammatory Tone in the Transition Period

The balance between inflammation and anti-inflammation is maintained through the profile of metabolites, oxylipins, cytokines, hormonal balance, and phenotype of immune cell [40,57,58]. The interaction between the various factors determines the maintenance of inflammation or the resolution of the inflammatory state with the establishment of tissue and systemic homeostasis. So, the increase in NEFAs, BHB, and consequent increase in ROS production in the postpartum period leads to the stimulation of inflammatory pathways through TLR, PPAR-γ (peroxisome proliferator-activated receptor gamma), and inflammasome, as in NLRP3, culminating in activation of NF-κB, increased cytokine production, and sustained inflammation [47,58]. This inflammatory state, driven by the metabolic environment, amplifies the inflammation and immunoreactivity observed at the end of pregnancy [4,39,49], but the maintenance of this state in the postpartum period disrupts tissue repair, which is essential for uterine involution and stimulates the occurrence of diseases [59,60].

**Figure 2:**
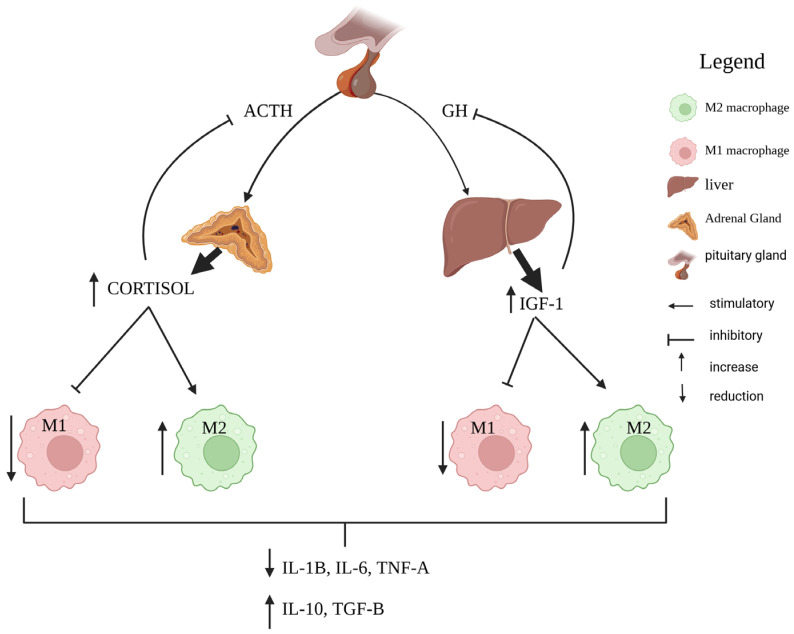
The present figure demonstrates immunologic and metabolic regulation of the immune system under physiological conditions. Growth hormone (GH) stimulates the production of insulin-like growth factor 1 (IGF-1), which acts in the regulation of metabolism and the profile of tissue macrophages, leading to an increase in M2 macrophages and anti-inflammatory cytokines (IL-10, TGF-β) and a reduction in M1 macrophages and inflammatory cytokines (IL-1β, IL-6, TNF-α). The release of adrenocorticotropic hormone (ACTH) stimulates the production of glucocorticoids (cortisol) by the adrenal gland, modulating the reduction in M1 macrophages and the increase in M2 macrophages. These hormones lead to an increase in M2 macrophages and a reduction in inflammatory cytokines, as well as an increase in anti-inflammatory cytokines, linked to the resolution of inflammation and the preservation of tissue homeostasis.

**Figure 3:**
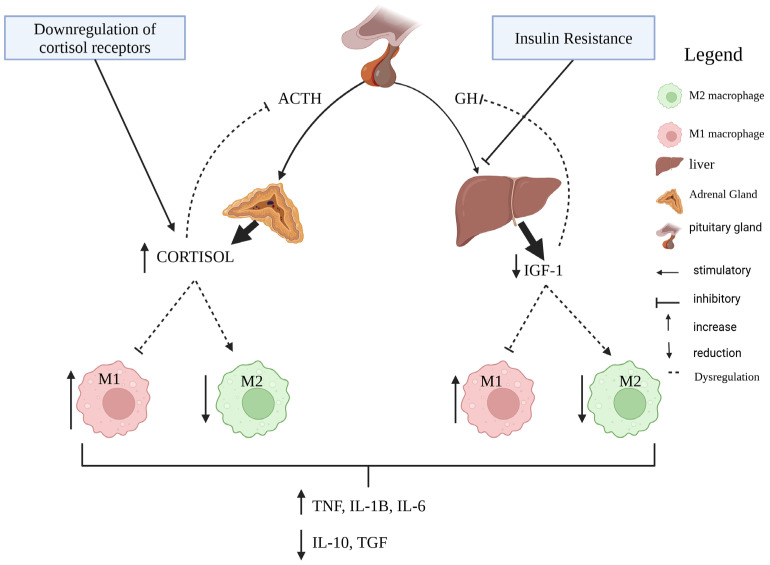
In the postpartum period, insulin resistance reduces the expression of GH receptors, reducing IGF-1 levels, which favors an increase in M1 macrophages and reduces M2. In turn, the reduction in cortisol sensitivity favors an increase in M1 macrophages. The reduction in cortisol responsiveness associated with reduced IGF-1 levels induces an increase in M1 macrophages, increasing the levels of inflammatory cytokines such as IL-1β, IL-6, and TNF-α, and reduces the levels of IL-10 and TGF-β, leading to inflammation and preventing inflammatory resolution.

The somatotropic axis plays a fundamental role in and is well known for the regulation of inflammatory tone, as shown in Figure 2 [14]. In the postpartum period, the uncoupling of the somatotropic axis and insulin resistance leads to a reduction in IGF-1 levels and intensifies lipolysis [12]. The reduction in IGF-1 levels is associated with metabolic changes in bovine leukocytes leading to aerobic glycolysis and inflammation [61]. Studies with other species have observed that anaerobic glycolysis causes maintenance of the macrophage profile in M1 and the development of chronic inflammation (Figure 3) [48,62], which for us demonstrates the interaction between the uncoupling of the somatotropic axis and the reduction in IGF-1 levels with the dysregulation of postpartum inflammatory resolution.

The hypothalamic–pituitary–adrenal axis and the maintenance of its responsiveness and sensitivity play a key role in maintaining the homeostasis of the innate immune system (Figure 3) [51]. Postpartum cows present elevated cortisol in blood, with a reduction in the pulsatility pattern of this hormone when stimulated with LPS [22,23], and depletion of the expression of glucocorticoid receptors in mononuclear leukocytes, demonstrating less responsiveness to the effects of this hormone [63], which reduces its anti-inflammatory effect (Figure 3). In humans, the role of cortisol resistance, ACTH levels, and their regulation in establishing the onset of the resolving phase of inflammation are well known [64,65], and we believe that this condition is relevant in dairy cows in the transition period where stress and inflammation occur, a hypothesis that needs to be confirmed by further research.

In the postpartum period, the reduction in NADPH levels due to its role in redox metabolism leads to a reduction in the synthesis of 13-HODE to 13-oxooctadecadienoic acid (13-oxoODE) due to the dependence of the dehydrogenase responsible for this reaction on NADPH [49,65]. The increase in ROS associated with the reduction in the activity of the NADPH-dependent dehydrogenase increases the synthesis of inflammatory oxylipins such as 13-HODE and 5-, 11-, and 15-HETE, reducing the levels of 13-oxoODE with an anti-inflammatory profile [38,40].

The study of inflammation resolution still requires further clarification in dairy cattle during the transition. In non-ruminants, the beginning of the inflammation resolution process involves the stimulation of ACTH release by inflammatory cytokines, reduction in chemokines and polymorphonuclear infiltration, neutrophil efferocytosis, change in the oxylipin profile from inflammatory to anti-inflammatory, and downregulation of inflammatory cytokine receptors, culminating in a phenotypic change in tissue macrophages from M1 to M2 [20,65]. Molecules, such as adenosine, and the action of the vagus nerve, with the release of acetylcholine, act to promote inflammatory resolution in humans [66], but the role of these axes in the regulation of inflammatory resolution is unclear in dairy cows.

Impaired inflammatory resolution and development of diseases in cattle are demonstrated during the transition. The relationship between adenosine deaminase, an enzyme involved in the control of adenosine levels, and calcium levels was observed, with an increase in the activity of this enzyme associated with a reduction in serum calcium [19]. Monocytes stimulated with fatty acids showed the development of an M1 profile associated with blockade of mammalian target of rapamycin (mTOR)-mediated autophagy [43] with a similar phenotype observed in macrophages from cows with subclinical ketosis [54], indicating impaired inflammatory resolution. Understanding the mechanism of inflammation resolution and how its dysregulation affects the dynamics of the onset of lactation can contribute to advances in knowledge of the etiopathogenesis of transition disorders.

For us, understanding the cholinergic and purinergic pathways in the regulation of the inflammatory and resolution process is key to modulating and enhancing dairy cow production. In other species, the activation of the alpha 7 nicotinic acetylcholine receptor (α7nAChR) by acetylcholine resulting from activation of the vagus nerve (vagal anti-inflammatory reflex) acts by reducing the expression of Cyclooxygenase 2 (COX2) and microsomal prostaglandin E synthase-1 (mPGES-1), culminating in a reduction in the levels of inflammatory cytokines and prostaglandins [66], but this mechanism has not been investigated in postpartum dairy cattle. This modulatory effect is vital for the initiation of the resolution of inflammation, since at high levels prostaglandin E2 (PGE) is inflammatory, and with the reduction in its levels, it presents anti-inflammatory effects, demonstrating the importance of down-modulation and not of blocking COX2 [66,67]. This mechanism was investigated in studies of LPS-induced bovine endometritis, and it was observed that α7nAChR agonists have a protective effect on the endometrium by promoting the reduction in COX2/mPGES-1 activity via inhibition of the Janus kinase 2 (JAK2)/signal transducer and activator of transcription 3 (STAT3) pathway [67]. In our view, modulation rather than blockade of COX2 may be a key element in ensuring healthy adaptation to lactation, maintaining high production, and preserving reproduction of dairy cows, requiring the study of new approaches for the transition and modulation of inflammation.

The purinergic system is categorized into two types: P1 receptors, which include adenosine receptors (ADORA) and have anti-inflammatory properties, and P2 receptors, which encompass nucleotide receptors (P2RY) and are associated with inflammatory responses [68,69]. In dairy cattle, an increase in the expression of adenosine A2a receptors and P2Y2 receptors in polymorphonuclear cells [69] is observed from 3 to 21 days postpartum, but in liver, a reduction in the expression of P1 receptors (ADORA2a and ADORA 3) and P2RX7 is observed, as well as increase in P2RX4 and P2RX11 [6]. However, the increase in the expression of the components of the purinergic system makes the cow more sensitive to its effects, but the postpartum phase is marked by disturbance of the energy status, and the maintenance of the levels of the molecules of the purinergic system (for example, adenosine) and their impacts are little known in the cow. The interaction of the purinergic system and the cholinergic system with mTORC 1 and 2 is a possibility in the transition, opening new pathways for interaction between immunity, metabolism, and the neuroendocrine system. However, research data on dairy cattle is still scarce.

Reversal of the pro-inflammatory state depends on the responsiveness of the hypothalamic–pituitary–adrenal axis associated with vagal activity, with increased ACTH/cortisol and acetylcholine, respectively. Adrenal hypo responsiveness, cortisol resistance, and low vagal tone are key conditions for failure to inflammation resolution and constitute a new paradigm in the understanding of inflammation in dairy cows. The resulting inflammatory state, of this condition, increases insulin resistance and disrupts the maintenance of cellular barrier integrity, resulting in exposure to LPS in inflammatory environment. This potentiates free radical production and may compromise the stability of cellular organelles, leading to pathological states. However, this hypothesis requires experimental confirmation.

## 4. Inflammation, Mitochondria Dysfunction, and Endoplasmic Reticulum Stress

With a central role in the production of ATP through oxidative phosphorylation, mitochondria act in the production of ROS, calcium homeostasis, thermogenesis, innate immune response, and apoptosis [70]. Mitochondrial dysfunction is associated with diseases such as mastitis [71], ketosis [72], and fatty liver [73] in dairy cattle. Studies including high-concentrate diets to induce an inflammatory state, and exposure to LPS demonstrated increased mitochondrial membrane permeability, reduced expression of genes related to mitochondrial fusion, and increased mitochondrial division, demonstrating a dysfunctional state of the mitochondria of mammary gland cells when in an inflammatory state [71]. The reduction in proteins involved in oxidative phosphorylation (OXPHOS) observed in cows with ketosis induces the “leakage” of electrons and their transfer to molecular oxygen, generating ROS [72]. The increase in mitochondrial ROS production associated with the activation of PPRs by LPS or mitochondrial DNA induces the activation of the NRLP3 inflammasome, increasing caspase 1, and induces activation by IL-1β cleavage [47,71]. Mitochondrial dysfunction disrupts the energy homeostasis of cells and plays a central role in the dysfunction of the immune response, with the energy generation pathway being an inducer of the profile of macrophages and cells of the immune system regarding inflammation and anti-inflammation [48,53]. Extending the role of mitochondrial dysfunction in inflammation in dairy cows increases the ability to understand and resolve transition diseases associated with inflammation.

The endoplasmic reticulum plays vital roles in protein synthesis, folding, and processing, glycosylation, calcium homeostasis, and metabolism of various substances [74]. Stress on this organelle leads to the development of the unfolded protein response, which is associated with increased ROS production and activation of tree sensor proteins, protein kinase R-like ER kinase (PERK), inositol-requiring enzyme 1α (IRE1α), and activating transcription factor 6 (ATF6), which drive the unfolded protein response restoring homeostasis [75,76]. Endoplasmic reticulum stress has been observed in postpartum cattle, associated with inflammation [75], playing a vital role in the progression of more severe fatty liver disease in dairy cows [76] and in cows with clinical ketosis [77]. Increased NEFAs and inflammation, together with increased endoplasmic reticulum stress, are observed in postpartum cows, with the effect of controlling inflammation or failure to resolve them remaining open questions about the genesis of endoplasmic reticulum stress in dairy cows. For us, the dichotomy between homeorhetic metabolism and deviation towards pathological states associated with transition diseases in dairy cows is represented by mitochondrial and endoplasmic reticulum function.

## 5. Inflammatory Modulation in Transition Period

The modulation of inflammation in dairy cows in the postpartum period through NF-κB blocking drugs (acetylsalicylic acid) or highly selective COX2 blockers (meloxicam) has demonstrated a sustained increase in milk production, reduction in ketogenesis, and increase in serum IGF-1 in cows in the immediate postpartum period [78,79,80]; however, their effects on the modulation of tissue resident cells are not clear in postpartum cows, and blocking cyclooxygenases with effects on PGE2 may be detrimental to the homeostasis of inflammatory resolution. Another challenge of these drugs is the period of milk withdrawal for human consumption due to its residual effects.

Phyto actives represent a possibility for modulating the challenges of inflammation in the transition, being able to regulate the inflammatory response and reduce oxidative stress [81]. Curcumin addition for dairy calves demonstrated marked effects on cytokines, with a reduction in IL-1β and IL-6 and an increase in IL-10, demonstrating a reduction in inflammation and an increase in anti-inflammation, with a reduction in lipid peroxidation being observed [82]. In mammary gland cells of cows exposed to LPS, curcumin reduced the accumulation of ROS and the expression of TNF-α, IL-8, IL-6, and IL-1β through the activation of nuclear factor erythroid 2-related factor 2 (Nrf2) [83]. Studies in humans demonstrate the effect of curcumin on the phenotype of tissue macrophages, with addition of this substance promoting an increase in M2 macrophages related to the resolution of inflammation and tissue homeostasis [84].

The addition of 25 g/d (0.1% of DMI) of *Scutellaria baicalensis* extract (flavonoids and baicalin) in the diet of canulated dairy cows has been shown to modulate the inflammatory response, with a reduction in TNF-α and IL-1β levels, a reduction in lipid oxidation, and an increase in antioxidant capacity [85]. The combined effect of essential oils (carvacrol, eugenol, cinnamaldehyde, and red pepper resin oil) were observed in cows in the final third of lactation, with a reduction in TNF-α, IL-1β, and IL-6, an increase in IL-10, a reduction in lipid peroxidation, and a depression in adenosine deaminase activity in lymphocytes, pointing to a positive effect on the resolution of inflammation. The use of the microencapsulated essential oil also modulated the activity of the cholinergic system, with a reduction in the activity of systemic cholinesterase and an increase in lymphocyte acetylcholinesterase, and an effect on the purinergic system was also observed with a reduction in the activity of lymphocyte adenosine deaminase [86].

The use of nutrition to modulate the immune system should be considered in the modulation of the inflammatory response in the transition. Studies on bovine immune system cells, in vitro-supplemented with choline, demonstrate increased expression of genes involved in the synthesis of choline-derived products together with genes responsible for muscarinic and nicotinic receptors in neutrophils and monocytes, showing quadratic behavior in relation to the choline dose [87]. These results contribute to the role of choline as an immunomodulatory agent through its metabolites and are also important in redox metabolism as a donor of methyl groups. The modulation of the inflammatory response in the transition is influenced by the lipid and amino acid profile of the diet. The balance of omega (ω) 3 and ω6 in the diet interferes with the substrates of omega hydrolases, altering the balance of inflammatory and anti-inflammatory oxylipins. Maintaining an ω6 to ω3 ratio between 3.9:1 and 5.9:1 is advisable [88]. Dietary concentrate levels have marked effects on inflammatory markers, and it has observed that diets with higher concentrate levels (>60%) increase oxidative stress with increased circulating LPS, compromising health maintenance [34,46]. Addition of adequate levels of metabolizable proteins with moderation of non-fibrous carbohydrate levels is recommended in cows during the transition period to maintain health.

## 6. Conclusions

Inflammation in dairy cows is a process inherent to calving and early lactation, but the ability to initiate inflammation resolution is a key determinant of health and a new paradigm. Low responsiveness of the hypothalamic–pituitary–adrenal axis and cortisol resistance disrupt the initiation of inflammatory resolution, maintaining chronic inflammation and elevating free radical levels, configuring the second paradigm. These factors catalyze the production of pro-inflammatory oxylipins and increased peripheral insulin resistance. Maintaining inflammation reduces dry matter intake and increases lipolysis, creating a vicious cycle.

The sustained inflammatory state associated with glycolysis disrupts the maintenance of energy homeostasis, which, combined with free radical production, leads to instability of the endoplasmic reticulum and mitochondria, allowing the onset of diseases such as fatty liver, thus determining the transition from the homeorhetic to the pathological state and configuring the third paradigm. The ability to maintain the purinergic system’s function is questionable due to the high metabolic demands of lactation, with acetylcholine levels resulting from vagal activity being disrupted by stress, thus compromising two additional pathways involved in inflammatory resolution, the fourth paradigm. Understanding the regulation of inflammatory resolution and the role of cholinergic and purinergic pathways associated with dysregulation of the hypothalamic–pituitary–adrenal axis constitutes a potential target for future research in the transition period of dairy cows.

The modulation of the inflammatory response demonstrates great potential for production, but challenges were observed regarding the use of conventional anti-inflammatories due to potential effects on the initiation of the inflammation resolution process, and the use of phytogenic substances as modulators of the inflammatory tone acting on the activity of the purinergic and cholinergic systems was also indicated, configuring a new approach and the fifth paradigm. We also point out the role of nutrition and omega 3 and 6 levels together with diets that are more friendly to gastrointestinal health, with additives such as choline, as factors in the modulation of immune health via the cholinergic system.

## Data Availability

The original contributions presented in this study are included in the article. Further inquiries can be directed to the corresponding author(s).
